# Influenza Vaccination Coverage and Intention to Receive Hypothetical Ebola and COVID-19 Vaccines among Medical Students

**DOI:** 10.3390/vaccines9070709

**Published:** 2021-06-30

**Authors:** Ewa Talarek, Joanna Warzecha, Marcin Banasiuk, Aleksandra Banaszkiewicz

**Affiliations:** 1 Department of Children’s Infectious Diseases, Medical University of Warsaw, 02-091 Warsaw, Poland; 2 University Children’s Hospital of Warsaw, 02-091 Warsaw, Poland; jm.sliwka@gmail.com; 3 Department of Pediatric Gastroenterology and Nutrition, Medical University of Warsaw, 02-091 Warsaw, Poland; mbanasiuk@wum.edu.pl (M.B.); abanaszkiewicz@wum.edu.pl (A.B.)

**Keywords:** influenza, vaccination, vaccine acceptance, COVID-19, Ebola, medical students

## Abstract

The study aimed to determine influenza vaccine uptake among medical students and their intention to receive a hypothetical Ebola or COVID-19 vaccine. This cross-sectional questionnaire-based study was performed in 2015 and 2020 on 675 medical students at the Medical University of Warsaw, Poland. In 2020, the influenza vaccination coverage was 36.5%, and students were almost five times more likely to be vaccinated than in 2015 (OR = 4.8; 95% CI: 3.1–7.5). In 2020, the survey was conducted during the first university campaign targeted at free influenza vaccinations for all students, as well as during the first lockdown in Poland due to the COVID-19 pandemic. In 2020, clinical students (4th–6th study year) were significantly more often vaccinated than preclinical students (*p* < 0.001), in contrast to 2015. A majority—67.0% and 94.6%—of students expressed their intention to receive a hypothetical Ebola or COVID-19 vaccine, respectively. Among the medical students, influenza vaccination status was a predictor of the intention to receive a COVID-19 vaccine. Influenza vaccine uptake among medical students has increased significantly, but it is still not optimal; thus, further educational efforts are needed to convince those who are hesitant regarding vaccines. A high number of students reported their intention to receive a COVID-19 vaccine, and it is crucial to support their positive attitude about it.

## 1. Introduction

Globally, seasonal influenza epidemics result in about 3–5 million cases of severe illness and about 250,000 to 500,000 deaths per year [1]. Annual influenza vaccination for everyone 6 months of age and older is strongly recommended by the Centers for Disease Control and Prevention (CDC) [2], the World Health Organization (WHO) [3], and many local authorities worldwide [4]. This recommendation is even stronger for health care workers (HCW), who are at high risk of transmitting infections to high-risk patients–e.g., young children, elderly people, pregnant women, and people who are immunocompromised or chronically ill [5,6].

Influenza vaccination rates are lower than the WHO’s 90% recommendation and there is considerable variability between countries. During the 2018–2019 influenza season, influenza vaccination coverage in the United States was 81.1% among HCWs, similar to coverage rates during the past four seasons (77.3% to 79.0%) [7]. Influenza vaccination coverage among HCWs is much lower in Europe, reaching only 14% in Italy [8] and 15% in Switzerland [9]. In Poland, only 4–5% of HCWs are vaccinated against influenza annually [10]. Knowledge of HCWs’ reasons for accepting or refusing vaccination is crucial in order to change attitudes and increase vaccination rates.

Medical students, having similar contact with patients as HCWs, are also an important target group for annual influenza vaccinations. If people are motivated to receive vaccinations as students, they are likely to continue being vaccinated as HCWs in the future [11]. Studies show that vaccination coverage among medical students ranges from 4.7% in Iran to 86.3% in Canada (in various seasons) [12,13,14,15,16,17,18,19,20,21]. Influenza vaccines are well-known, safe, and recommended by medical authorities, yet only a fraction of Polish medical students decide to be vaccinated. Vaccination coverage among Polish medical students was only 14% in the 2012–2013 season [22].

The aim of the study was to determine influenza vaccine uptake among Warsaw medical students and to ascertain their reasons for accepting or refusing vaccination. In Poland, influenza vaccination is not reimbursed for medical students. From February until April 2020, influenza vaccination was available free of charge for all students at the Medical University of Warsaw, regardless of study year. It was the first such campaign at the University. The announcement was published and repeated on the University website, and vaccination was conducted on the University campus.

Additionally, we assessed the attitudes of medical students towards immunization with a new (hypothetical) vaccine that would control an epidemic, and whether seasonal influenza vaccination predicted their intentions to receive this “epidemic” vaccine. We performed our evaluation twice: once in the winter of 2014–2015 and again in the spring of 2020. In 2014–2015, the epidemic of Ebola haemorrhagic fever was ongoing in West Africa. There was a low risk of Ebola spread outside of Africa, but singular imported cases occurred in the United States and Europe (though not in Poland) [23,24]. In March 2020, the WHO declared a pandemic of a coronavirus disease (COVID-19) caused by a novel coronavirus, severe acute respiratory syndrome coronavirus 2 (SARS-CoV-2). The first lockdown in Poland began on 20 March 2020, before the survey was conducted. At the moment of the survey, schools and universities were closed, as well as cultural facilities, shopping centers, restaurants etc., there were limits of people in the shops and public transport. In April 2020, in Poland there were 237–475 cases of SARS-CoV-2 infection confirmed daily, reaching a total number of 12,877 cases on the 30th April.

From the beginning of this pandemic, scientists started work on developing a vaccine against COVID-19; it is expected that immunization will be a major measure in containing the pandemic.

## 2. Material and Methods

A cross-sectional survey study was conducted in April and June of 2015 and in March and April of 2020. Students at the Medical University of Warsaw in Poland were asked to complete an anonymous questionnaire. In 2015, the questionnaires were administered in paper form to students in the first and sixth (final) study year during lectures and clinical classes. In 2020, a link to an electronic version of the survey was sent by email to all students from the first to the final year.

The questionnaire consisted of three parts: (i) demographic questions (age, sex, and study year); (ii) questions concerning influenza vaccination status in the current season (2014–2015 or 2019–2020) and reasons for receiving or refusing the vaccine; and (iii) questions concerning intention to be vaccinated against Ebola (in 2015) or COVID-19 (in 2020).

Distribution of data was evaluated by Shapiro–Wilk and Kolmogorov–Smirnoff tests. Continuous variables were expressed as median and interquartile range (IQR). Categorical variables were compared using the Chi-square test with Yates’s correction or the Fisher exact test, when appropriate. A 95% confidence interval (CI) was calculated to reveal the significance of odds ratio (OR). Statistical tests were performed using Statistica 13 (Tibco Software Inc., Palo Alto, CA, USA). *p*-values < 0.05 were considered statistically significant.

## 3. Results

**Figure 1:**
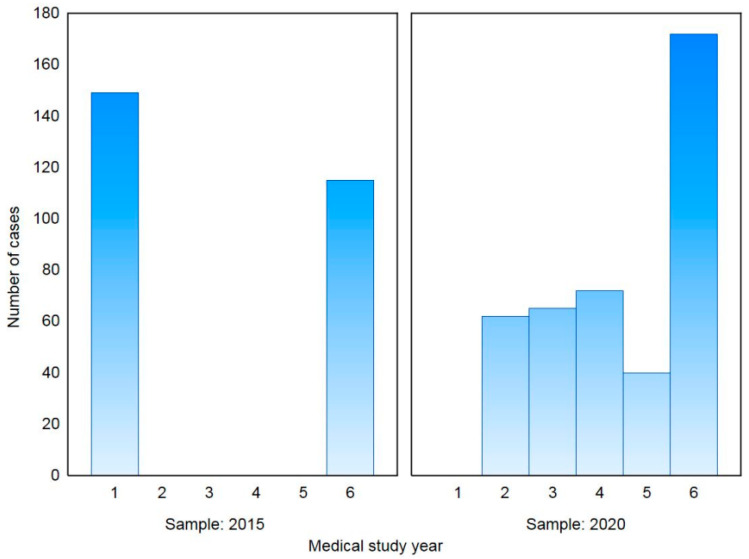
Distribution of medical study year in the 2015 and 2020 samples.

The 2015 sample consisted of 264 medical students, with 100 (37.9%) males and a median age of 21 years (range: 20–25); there were 149 students in the first year and 115 students in their sixth year of study. The 2020 sample consisted of 411 medical students, with 130 (31.6%) and a median age of 24 years (range: 22–25 years); there were 127 and 284 students in the 2nd–3rd and 4th–6th study year, respectively. Demographic characteristics and influenza vaccination status of both samples and all subjects are presented in Table 1. Distribution of medical study year, in both 2015 and 2020 samples, is shown in Figure 1. For further analysis, a division for preclinical students (1st–3rd year of medical study) and clinical students (4th–6th year of medical study) was used. The samples varied; in 2015, there were significantly more preclinical students while, in 2020, there were significantly more clinical students (*p* < 0.001).

There was a statistically significant difference in influenza vaccination status between medical students in 2015 and 2020 (Table 2). In 2020, students were almost five times more likely to be vaccinated than in 2015 (OR = 4.8; 95%CI: 3.1, 7.5).

**Figure 2:**
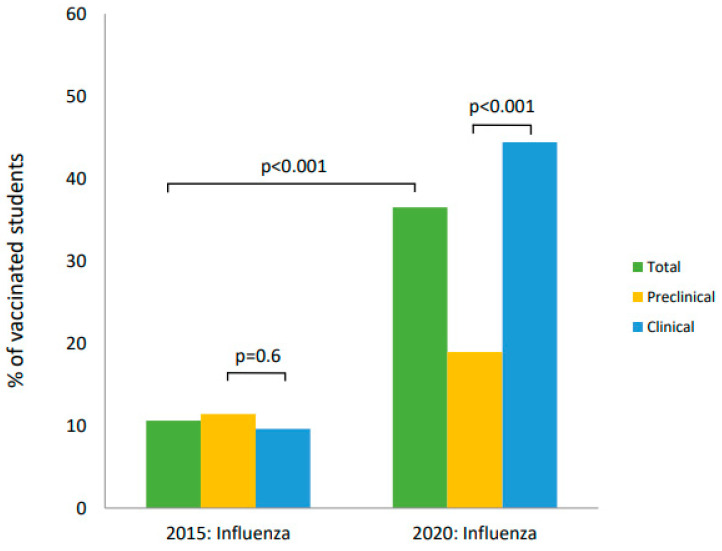
Influenza vaccination coverage among medical students in 2015 and 2020.

In 2015, preclinical students were more often vaccinated than clinical students, but the difference was not statistically significant (*p* = 0.629). However, in 2020, clinical students received influenza vaccinations significantly more willingly (*p* < 0.001). Proportions of vaccinated students in 2015 and 2020 are shown in Figure 2.

Table 3 shows medical students’ reasons for receiving or not receiving a vaccination. The most common reason for being vaccinated varied across the two samples. In 2015, 57.1% of vaccinated students reported fear of contracting the disease as their main reason for vaccination. In 2020, however, vaccinated students most often reported belief in vaccine efficacy.

There was a statistically significant difference in the proportion of vaccinated students who declared a belief in vaccine efficacy between 2015 and 2020: 39.3% vs. 88%, respectively (*p* < 0.001). The most common reason for not being vaccinated was the same in both 2015 and 2020: not being afraid of contracting influenza. However, this reason was less frequently reported in 2020 than in 2015 (44.1% vs. 54.7%, respectively; *p* = 0.020). Financial reasons for not being vaccinated were significantly more frequent in 2020 than in 2015 (33% vs. 7.2%, respectively; *p* < 0.001).

A majority of students reported that they would agree to be vaccinated if a vaccine against Ebola (2015) or COVID-19 (2020) was available: 67% and 94.6%, respectively (*p* < 0.001). Intention to receive a hypothetical Ebola vaccine is presented in Table 4, and intention to receive a hypothetical COVID-19 vaccine is presented in Table 5. In 2015, acceptance was significantly more common among preclinical students than clinical students (76.5% vs. 54.8%, respectively, *p* < 0.001). In 2020, there was no difference between preclinical and clinical students, and almost all of them would accept a COVID-19 vaccine.

If a vaccine against Ebola or COVID-19 caused pain or fever, it would be accepted less often (56.1% vs. 86.6%, respectively; *p* < 0.001), but even with these side effects, students in 2020 were five times more likely to decide to be vaccinated against COVID-19 than students in 2015 were to be vaccinated against Ebola (OR = 5; 95% CI: 3.5–7.4). If a vaccine against Ebola or COVID-19 had moderate efficacy (50%), a lower proportion of medical students would opt to be vaccinated (51.1% vs. 83%, respectively; *p* < 0.001). In both 2015 and 2020, there was no statistically difference in the acceptance of a hypothetical vaccine between preclinical and clinical students if it caused local pain and fever (*p* = 0.034 and *p* = 0.060, respectively) or had 50% efficacy (*p* = 0. 526 and *p* = 0.455, respectively).

In 2015, influenza vaccination status was not associated with the intention to be vaccinated against Ebola (OR = 1.5; 95% CI: 0.6–3.8; *p* = 0.3), as shown in Table 6. Preclinical students more often reported an intention to be vaccinated against Ebola, but the difference was only statistically significant among those non-vaccinated against influenza.

Hypothetical vaccination against Ebola was accepted significantly more often among students who were already vaccinated for influenza vs. those who were not vaccinated for influenza, but this was only the case in a moderately efficacious Ebola vaccine (71.4% vs. 48.7%, respectively; *p* = 0.020). Influenza vaccination status was a predictor of intention to be vaccinated against COVID-19 in 2020 (Table 7). Students vaccinated against influenza were more likely to accept a hypothetical COVID-19 vaccine in general (OR = 6.1; 95%CI: 1.4, 26.6; *p* = 0.006), even in the case of vaccine side effects (OR = 4.6; 95%CI: 2.0, 10.5; *p* < 0.001) or a 50% efficacy rate (OR = 2.2; 95%CI: 1.2, 3.9; *p* = 0.009). There were no differences between preclinical and clinical students.

## 4. Discussion

In this cross-sectional study, we found that a significantly higher proportion of medical students reported influenza vaccination in 2019–2020 than in 2014–2015 (36.5% vs. 10.6%, respectively). Among clinical students, the increase was even more significant (9.6% vs. 44.4%, respectively). Although this apparent increase in the vaccination rate among medical students is encouraging, the vaccination rate itself remains insufficient. Previous studies investigating influenza vaccination coverage among medical students worldwide reported vaccination rates varying from 4.7% in Iran to 86.3% in Canada [12,13,14,15,16,17,18,19,20,21]. Some studies found that clinical medical students were more likely to be vaccinated than those still in pre-clinical education [17,19], but this was not confirmed by subsequent investigations [13,25]. In this study, influenza vaccination coverage among preclinical and clinical students varied; in 2015, it was significantly higher in preclinical students and in 2020 it was significantly higher in clinical students. The last finding may be explained by clinical students’ awareness of influenza and recommendations for annual vaccination, as well as their compliance with those recommendations.

We can speculate that information publicized in the mass media regarding the potential protective effect of influenza vaccination against COVID-19 may be one of the reasons underlying the increased influenza vaccination rate among medical students in 2020. There was also a media message in 2020 that influenza symptoms and COVID-19 symptoms are similar, and protection against influenza through vaccination may decrease the number of misleading cases. Additionally, in February–April 2020, students at the Medical University of Warsaw were vaccinated against influenza for free. Lack of cost and easy access to vaccination on the University campus could explain, at least partially, significant increases in vaccination coverage among medical students in 2020. It is worth noting that the campaign was rather late in the influenza season, and the effect would probably have been even better if the vaccinations were offered earlier.

Previous studies concerning influenza vaccination among medical students have also examined the reasons for accepting or refusing vaccination. Reasons for acceptance were mainly self-protection, patient protection, and availability of vaccinations free of charge. In the current study, the main reason for receiving the influenza vaccine varied between two samples: in 2015, it was fear of the disease (which results a desire for self-protection); in 2020, it was belief in vaccine effectiveness. Interestingly, more than two-thirds of students in 2020 indicated fear of influenza complications as a reason for being vaccinated. Students are young and mostly otherwise healthy, so the fear of influenza complications may indicate that their knowledge about the disease is not accurate, since they are generally not at risk of a severe or complicated clinical course of influenza. Our study uncovered one rather depressing finding: in both 2015 and 2020, only a small proportion of students (21.4% and 26%, respectively) reported being vaccinated against influenza due to their doctor’s recommendation. There may be few explanations for this. First, students may not have a family doctor in the city where they attend school and may not visit a family doctor while in medical school. Second, family doctors may not advise annual influenza vaccination or may even discourage it. Third, medical students may not consider family doctors an authority and therefore do not heed their advice. A similar proportion of Australian medical students (21.1%) reported a doctor’s recommendation as a motivator for influenza vaccination [19].

Reasons for vaccine refusal in previously cited studies were inconvenience, forgetfulness, low risk perception, fear of side effects, and disbelief in vaccine effectiveness. Here, the most common reason for not being vaccinated was low risk perception (i.e., “I’m not afraid to get flu”). The next most common reasons were disbelief in vaccine effectiveness (2015) and the cost of a vaccine (2020). Interestingly, the proportion of students doubting vaccine effectiveness almost halved from 2015 to 2020: only 10% of those who refused vaccination cited this as an explanation in 2020 (19.55% in 2015). An even smaller proportion refused vaccination in the 2019–2020 season because of concerns over vaccine side effects. Therefore, it seems that influenza vaccines are perceived as effective and safe, even by medical students who refuse them. It may therefore be more important to educate students about influenza itself rather than about vaccination, emphasizing that influenza poses a serious health risk for certain patients and there is an ethical imperative to protect them from infection. Knowledge of barriers and addressing them in education and strategic activities are crucial to convince “vaccine hesitant” individuals [26,27].

Vaccination costs have been reported by others as a barrier to vaccine uptake [16] and, in our 2020 sample, one-third of students cited this as a reason for refusing vaccination. This represented a statistically significant increase in comparison to the 2015 sample (7.2%, *p* < 0.001). As mentioned, influenza vaccinations are not reimbursed for medical students in Poland, and only some healthcare facilities vaccinate their personnel for free. The cost of the influenza vaccine is relatively low but, in the 2019–2020 season, it was almost twice as costly (approximately €10) as in the 2014–2015 season. When the 2020 survey was conducted, influenza vaccination was offered free of charge to students of the Medical University in Warsaw, but it was an unprecedented situation.

Our study also assessed medical students’ intentions to receive a hypothetical vaccine against an epidemic disease—Ebola in 2015 and COVID-19 in 2020—and the association between this intention and influenza vaccination status. In the 2015 sample, two-thirds of the students declared an intention to accept a hypothetical Ebola vaccine. A majority of students declared that they would accept a vaccine even if it were only 50% effective or would cause fever or local pain. There was a statistically significant difference in the opinions of students who were vaccinated against influenza and those who were not (*p* = 0.02) concerning the hypothetical scenario of an Ebola vaccine with only 50% effectiveness. The proportion of the students declaring an intention to be vaccinated against Ebola (67%) was surprisingly high, considering that Ebola was only hypothetical threat in Poland in 2015. In a previous study conducted among the general population in the US (where a few cases of Ebola occurred), one-third of participants expressed interest in taking an Ebola vaccine [28]. In Germany, this proportion was <20% [29]. Willingness to be vaccinated against the Ebola virus can be explained by the perception of Ebola as a severe disease with a high mortality rate and a new threat, even if the actual risk of contracting the disease is quite low.

At the time the 2020 survey was conducted, the risk of an epidemic disease was much higher than the first survey in 2015. COVID-19 had already been declared a pandemic by the WHO and, although local transmission in Poland was lower than in some other European countries, the Polish government enforced widespread lockdowns in order to slow the spread of COVID-19. These circumstances may explain why most the students surveyed in 2020 declared a willingness to take a hypothetical COVID-19 vaccine, even if it were only 50% effective or would cause fever and local pain. The role of social and mass media is noteworthy here. From the beginning of the COVID-19 pandemic, the media has perpetuated the message that only mass immunization with a vaccine against COVID-19 can stop the spread of the disease. As Lehmann et al. [15]) demonstrated, intention to be vaccinated does not necessarily mean that people will be vaccinated. They found an intention–behavior gap: only half as many students were vaccinated against influenza as those who declared their intention to get vaccinated [15]. Therefore, it is essential to identify barriers to vaccination, both objective (e.g., cost, availability, and ease of access) and psychological. Barello et al [30] reported that 86.1% of Italian students (mainly from the Lombardy region) intended to get vaccinated against COVID-19, and there was no significant difference between healthcare students and non-healthcare students. The lower rate of intention to receive a COVID-19 vaccine than in our study is a surprise, as Italy, especially Lombardy, was hit extremely hard by the first wave of the COVID-19 pandemic (March–May 2020). In a U.S., the study proportion of vaccine-hesitant medical students was 23% [31]. It seems that the perception of danger may be more important than the danger itself. It works even in relation to infectious disease in healthcare workers, such as medical students. Similarly, surveys conducted in the general population of the US, seven European countries, and China have indicated widespread willingness to be vaccinated against COVID-19 in 57.6%, 62.0–80.0%, and 91.3% of respondents, respectively [32,33,34]. Once again, intention to be vaccinated can differ from actual vaccination uptake rates. This depends on many factors, among which the cost of a vaccine and ease of access are probably not the most important. Available data indicate that a large enough proportion of vaccine sceptics in the US and Europe could make it impossible to achieve the herd immunity threshold, which was estimated at 74% for Europe [33].

We found that seasonal influenza vaccination status was a predictor of acceptance for COVID-19 vaccination, but not of Ebola vaccination. The proportion of students declaring intention to receive a COVID-19 vaccine was significantly higher among those who were already vaccinated against influenza than those who were not. This can mean that people who receive the seasonal influenza vaccine are, in general, “pro” vaccination for other infections such as COVID-19.

### Limitations

A potential limitation is that the respondents (i.e., students from one medical university) are not representative of all medical students. Participation in the study was voluntary, and students who participated may be more “pro” vaccination. Finally, response options for questions about accepting vs. refusing an influenza vaccine were limited to reduce the length of the questionnaire, and therefore may not have captured the full range of student opinions.

## 5. Conclusions

Influenza vaccination coverage among Warsaw medical students in the 2019–2020 season was much higher than in previous years, but it is still not optimal. An extremely high proportion of students expressed the intention to receive vaccination against COVID-19, and current influenza vaccination status was a predictor of acceptance for a COVID-19 vaccine. In this moment, we face the influenza season in conjunction with the second wave of the pandemic. COVID-19 immunization has been launched, so both education and effective public health messaging are needed to increase influenza vaccine uptake and to support positive attitudes toward COVID-19 vaccination among medical students.

## Figures and Tables

**Table 1:** Characteristics of study participants.

Sample	Year 2015 (*n* = 264)	Year 2020 (*n* = 411)
Year of study (median, IQR)	1 (1–6)	5 (2–6)
Age (median, IQR)	21 (20–25)	24 (22–25)
Male gender (n, %)	100 (37.9%)	130 (31.6%)
Total sample	Vaccinated against influenza (*n* = 178)	Unvaccinated against influenza (*n* = 497)
Year of study (median, IQR)	5 (4–6)	4 (1–6)
Age (median, IQR)	24 (22–25)	23 (21–25)
Male gender (n, %)	63 (35.4%)	167 (33.6%)

**Table 2:** Influenza vaccination status of medical students in 2015 and 2020.

Medical Students	Year 2015			Year 2020			*p*-Value
Total vaccinated	28/264 (10.6%)			150/411 (36.5%)			<0.001
Total non-vaccinated	236/264 (89.4%)			261/411 (63.5%)			
Preclinical	149/264 (56.4%)			127/411 (30.9%)			<0.001
Clinical	115/264 (43.6%)			284/411 (69.1%)			
Students	Preclinical	Clinical	*p*-value	Preclinical	Clinical	*p*-value	
Vaccinated	17/149 (11.4%)	11/115 (9.6%)	0.629	24/127 (5.8%)	126/284 (30.7%)	<0.001	
Non-vaccinated	132/149 (88.6%)	104/115 (90.4%)		103/127 (81.1%)	158/284 (33.6%)		

**Table 3:** Reasons for the decision to be vaccinated against influenza.

Sample	2015 (*n* = 264)	2020 (*n* = 411)	*p*-Value
Positive decision			
I do not want to get flu	16 (57.1%)	105 (70%)	0.200
I’m afraid of flu complications	9 (32.1%)	104 (69.3%)	<0.001
I believe in vaccine efficacy	11 (39.3%)	132 (88%)	<0.001
My doctor recommends influenza vaccination	6 (21.4%)	39 (26%)	0.600
Negative decision			
I’m not afraid to get flu	129 (54.7%)	115 (44.1%)	0.020
I’m afraid of vaccine side effects	10 (4.2%)	15 (5.7%)	0.400
I’m afraid of injections	3 (1.3%)	15 (5.7%)	0.008
I don’t believe in vaccine efficacy	46 (19.5%)	26 (10%)	0.003
My doctor doesn’t recommend influenza vaccination	11 (4.7%)	19 (7.3%)	0.200
I don’t have money for vaccination	17 (7.2%)	86 (33%)	<0.001

**Table 4:** Intention to get a hypothetical Ebola vaccine in 2015.

Ebola Vaccine	Total	Preclinical Students	Clinical Students	*p*-Value
If a vaccine was available				
I would get it	177/264 (67.0%)	114/149 (76.5%)	63/115 (54.8%)	<0.001
I would not get it	87/264 (33.0%)	35/149 (23.5%)	52/115 (45.2%)	
If a vaccine caused pain or fever after injection				
I would get it	148/264 (56.1%)	92/149 (61.7%)	56/115 (48.7%)	0.034
I would not get it	116/264 (43.9%)	57/149 (38.3%)	59/115 (51.3%)	
If a vaccine efficacy was moderate (50%)				
I would get it	135/264 (51.1%)	84/149 (56.4%)	51/115 (44.3%)	0.526
I would not get it	129/264 (48.9%)	65/149 (43.6%)	64/115 (55.7%)	

**Table 5:** Intention to get a hypothetical COVID-19 vaccine in 2020.

Intention to Get A COVID-19 Vaccine	Total	Preclinical Students	Clinical Students	*p*-Value
If a vaccine was available				
I would get it	389/411 (94.6%)	120/127 (94.5%)	269/284 (94.7%)	0.924
I would not get it	22/411 (5.4%)	7/127 (5.5%)	15/284 (5.3%)	
If a vaccine caused pain or fever after injection				
I would get it	356/411 (56.1%)	104/127 (81.9%)	252/284 (88.7%)	0.060
I would not get it	116/411 (43.9%)	23/127 (18.1%)	32/284 (11.3%)	
If a vaccine efficacy was moderate (50%)				
I would get it	341/411 (83.0%)	108/127 (85.0%)	233/284 (82.0%)	0.455
I would not get it	70/411 (17.0%)	19/127 (15.0%)	51/284 (18.0%)	

**Table 6:** Influenza vaccination status and intention to be vaccinated against Ebola in 2015.

	Influenza Vaccination Status	Vaccinated (*n* = 28)	Non-Vaccinated (*n* = 236)	*p*-Value
I Would Get Vaccinated against Ebola				
If a vaccine was available		Total: 21/28 (75.0%)	Total: 156/236 (66.1%)	0.300
		Preclinical: 13/17 (76,5%)	Preclinical: 101/132 (76.5)	
		Clinical: 8/11 (72,7%)	Clinical: 55/104 (52.9%)	
If a vaccine caused pain and fever after injection		Total: 16/28 (57.1%)	Total: 132/236 (56.1%)	0.900
		Preclinical: 9/17 (52.9%)	Preclinical: 83/132 (62.9%)	
		Clinical: 7/11 (63.6%)	Clinical: 49/104 (47.1%)	
If a vaccine efficacy was moderate (50%)		Total: 20/28 (71.4%)	Total: 115/236 (48.7%)	0.020
		Preclinical: 12/17 (70.6%)	Preclinical: 72/132 (54.5%)	
		Clinical: 8/11 (72.7%)	Clinical: 43/104 (44.1%)	

**Table 7:** Influenza vaccination status and intention to be vaccinated against COVID-19 in 2020.

	Influenza Vaccination Status	Vaccinated (*n* = 150)	Non-vaccinated (*n* = 261)	*p*-Value
I Would Get Vaccinated against Ebola				
If a vaccine was available		Total: 148/150 (98.7%)	Total: 241/261 (92.3%)	0.006
		Preclinical: 24/24 (100.0%)	Preclinical: 96/103 (93.2%)	
		Clinical: 124/126 (98.4%)	Clinical: 145/158 (91.8%)	
If a vaccine caused pain and fever after injection		Total: 143/150 (95.3%)	Total: 213/261 (81.6%)	<0.001
		Preclinical: 24/24 (100.0%)	Preclinical: 80/103 (77.7%)	
		Clinical: 119/126 (94.4%)	Clinical: 133/158 (84.2%)	
If a vaccine efficacy was moderate (50%)		Total: 134/150 (89.3%)	Total: 207/261 (79.3%)	0.009
		Preclinical: 23/24 (95.8%)	Preclinical: 85/103 (82.5%)	
		Clinical: 111/126 (88.1%)	Clinical: 122/158 (77.2%)	

## Data Availability

The data presented in this study are available on request from the corresponding author.
